# Using Biotechnology to Solve Engineering Problems: Non-Destructive Testing of Microfabrication Components

**DOI:** 10.3390/ma10070788

**Published:** 2017-07-12

**Authors:** Carla C. C. R. de Carvalho, Patrick L. Inácio, Rosa M. Miranda, Telmo G. Santos

**Affiliations:** 1 iBB-Institute for Bioengineering and Biosciences, Department of Bioengineering, Instituto Superior Técnico, Universidade de Lisboa, Av. Rovisco Pais, 1049-001 Lisbon, Portugal; 2 UNIDEMI, Department of Mechanical and Industrial Engineering, NOVA School of Science and Technology, NOVA University Lisbon, 2829-516 Caparica, Portugal; p.inacio@campus.fct.unl.pt (P.L.I.); rmmdm@fct.unl.pt (R.M.M.); telmo.santos@fct.unl.pt (T.G.S.)

**Keywords:** micro defects, NDT, indentation, microfabrication, *Staphylococcus*, *Rhodococcus*

## Abstract

In an increasingly miniaturised technological world, non-destructive testing (NDT) methodologies able to detect defects at the micro scale are necessary to prevent failures. Although several existing methods allow the detection of defects at that scale, their application may be hindered by the small size of the samples to examine. In this study, the application of bacterial cells to help the detection of fissures, cracks, and voids on the surface of metals is proposed. The application of magnetic and electric fields after deposition of the cells ensured the distribution of the cells over the entire surfaces and helped the penetration of the cells inside the defects. The use of fluorophores to stain the cells allowed their visualisation and the identification of the defects. Furthermore, the size and zeta potential of the cells and their production of siderophores and biosurfactants could be influenced to detect smaller defects. Micro and nano surface defects made in aluminium, steel, and copper alloys could be readily identified by two *Staphylococcus* strains and *Rhodococcus erythropolis* cells.

## 1. Introduction

Current developments in microfabrication are demanding for reliable, economical, and ecologic non-destructive testing (NDT) techniques to detect unprecedented micro defects that conventional NDT cannot perceive. Usual defects in microfabrication are roughness, surface micro cracks, or voids created by micro size particle detachments, such as those observed e.g., in powder micro-injection [1]. New engineering materials such as nanostructured materials, functional surfaces and thermal barrier coatings, microelectronic and optical components, biomedical and orthodontic devices, and solar cells, may have their efficiency and reliability dependent on the existence of microcracks. These micro size surface defects are particularly critical, as it is there that the mechanical stresses are more intense, fatigue starts, and external damage (corrosion, wear, and thermal effects) exist.

Conventional NDT techniques include: (i) the eddy current technique, in which spatial resolution and size of the samples that may be analysed depend on the dimensions of the coil used [2,3]; (ii) optical transmission, by which the cracks that may be passed through by a broad-spectrum light or laser diode are detected by a CCD camera whilst those with odd shapes through which light cannot be transmitted will not be visible [4]; (iii) radiography and tomography, high-cost NDT methods, have difficulties in detecting defects smaller than 2% the thickness of the material, those perpendicular to the radiation beam, and defects in parts of complex three-dimensional geometry due to difficulties in image interpretation [5,6]; (iv) dye penetrant testing, in which the volume of the defect is more important than its area, because the actual crack depth is strongly related to the probability of detection [7]; and, magnetic particle inspection which may be used for the detection of defects on ferromagnetic materials [8]. Although most of these techniques are useful for the detection of defects during traditional manufacturing, they are no longer appropriate for microfabrication, as they were developed to answer other requirements and target scenarios, involving different materials, defect sizes, and morphologies. In micro-manufacturing, component size is small, preventing an effective coupling of ultrasound or eddy current probes. Dye penetrant testing allows the identification of some defects with ≈0.9 µm, but only when the defect depth is large as compared to its superficial open area, since its physical principle is exclusively based on the high capillarity and low viscosity of the penetrant. Magnetic particle inspection cannot be applied to most engineering materials, including polymers, ceramics, composites, and metals such as aluminium, copper, titanium, magnesium, and stainless steels alloys. Besides, parts with complex geometries may present areas with little or no magnetic flux [8].

The limit of detectability and sensitivity of conventional NDT techniques have improved during the last decades, but defects on the micrometre scale are still difficult to identify over background noise [9]. Several sophisticated characterisation techniques for microfabrication are available, such as Scanning Acoustic Microscopy (SAM), Near-field Scanning Optic Microscopy (MSOM), Scanning Electron Microscope (SEM) or Atomic Force Microscopy (AFM). However, they are limited to very small planar areas, are expensive, and require considerable time for analysis and interpretation of images [4,10], targeting a different scenario compared to our envisaged applications.

We have recently proposed a novel approach for the detection of micro defects and material characterisation based on the use of bacterial cells stained with fluorescent dyes [11,12]. The initial validation tests showed that *Rhodococcus erythropolis* cells allow the identification defects with a depth of 6.8, 4.3, and 2.9 µm in copper, aluminium, and steel [11]. The application of electric and magnetic fields to the samples when using *R. erythropolis* and *Staphylococcus* sp. cells, respectively, improved the movement of the cells over the surfaces which resulted in the observation of defects made by nanoindentation, and during micro-powder injection moulding and micro-laser welding [12]. However, the cells in the previous studies presented their intrinsic properties and no further exploitation of their ability to produce specialized compounds or to adapt to certain conditions was made.

In the present study, the properties of bacterial cells were influenced to improve the interactions with surfaces and the detectability threshold of small defects. By influencing the net surface charge of the cells and the natural production of biosurfactants, it was possible to help their penetration into micro-scale defects. The micro-indentations for the tests were made with side-lengths below the detection limit of eddy current techniques (ca. 50 µm [13]), with a very low ratio of depth/open area (unfavourable for dye penetrant testing), and on samples thicker than those that could be examined by radiography, tomography, and optical transmission techniques. Our study is an example of the advantages of multidisciplinary approaches to solve real problems created by new manufacturing techniques.

## 2. Results

### 2.1. Identifying Suitable Bacteria for NDT 

To better observe micro-defects, the stained bacterial cells, which behave like solid particles in liquid suspensions, should cover the whole surface to be analysed and be able to enter and remain in the depressions. The application of cellular properties such as net surface charge, production of biosurfactants that decrease the surface tension of the liquid medium, and the production of siderophores to capture iron ions to increase the interaction between cell and magnetic and electric fields could represent a significant benefit when compared to the application of inert particles in NDT. Bacterial cells have evolved to form biofilms on the surface of most of the surfaces on Earth and bacterium–surface interactions involve electrostatic attraction or repulsion, Lifshitz-van der Waals and Brownian motion forces and Lewis acid–base interactions as described by the extended Derjaguin, Landau, Verwey, and Overbeek (xDLVO) theory [14,15].

Initially, we found that *Rhodococcus erythropolis* DCL14 moved in response to the direction of the applied electric field but no bacterium from our collection responded to magnetic fields. The first stage of this study was thus the screening and isolation of bacterial strains able to move as response to magnetic fields and the definition of the culture conditions leading to an increased electric/magnetic character of the cells. To favour the detection of defects in metallic surfaces, bacterial cells that could be influenced by magnetic or electric fields were sought in the space between the battery and the electronic board of cell phones. Since radio and microwave frequency electromagnetic fields (EMF) are emitted by mobile phones, bacterial cells living attached to these devices were expected to be influenced by EMF. Most of the aerobic bacterial species isolated using cultivation-based techniques from samples collected in the mobile phones were identified by the Sherlock^®^ Microbial ID System from MIDI, Inc., (Newark, DE, USA) as belonging to the genus *Bacillus* and *Staphylococcus* (each represented ca. 40.0% of the total population; data not shown). These cells have been found in mobile phones by other research groups [16,17].

All isolated strains from the mobile phones were tested for their ability to respond to magnetic fields. Of the six strains that followed the movement of magnets under the microscope, two *Staphylococcus* strains were, surprisingly, the most responsive and were chosen for the following studies. Under the conditions used for growth, these *Staphylococcus* cells were spherical, their diameter ranging between 0.52 and 0.72 µm. One of the *Staphylococcus* isolates, designated “SA”, shared a very high homology with several strains of *S. aureus*. The 16S rRNA-encoding gene sequence was aligned and found to be 100% identical to the 16S rRNA-encoding genes of *S. aureus* strains deposited in the GenBank database (data not shown). Strain “SA” was also identified as *S. aureus* by the Sherlock^®^ Microbial ID System, and will therefore be designated as *S. aureus* SA in the following sections of this manuscript. The other *Staphylococcus* strain that responded to magnetic fields, which was designated as “SH”, exhibited a sequence similar to that of the 16S rRNA-encoding gene of several other strains of the *Staphylococcus* genus. A homology of 99% was found between strain “SH” and *Staphylococcus* sp. HB2 (data not shown), which was isolated in China, where the mobile phone from which the SH strain was isolated was manufactured. Strain SH presented the highest similarity index with *S. hominis* in the Sherlock^®^ Microbial ID System, but similarities of 98% were obtained with 16S rRNA-encoding gene sequences of different *Staphylococcus* species and thus this strain isolated from the mobile phone will be referred to as *Staphylococcus* sp. SH from now on.

### 2.2. Influencing Bacterial Properties to Help Cell Adhesion

**Figure 1:**
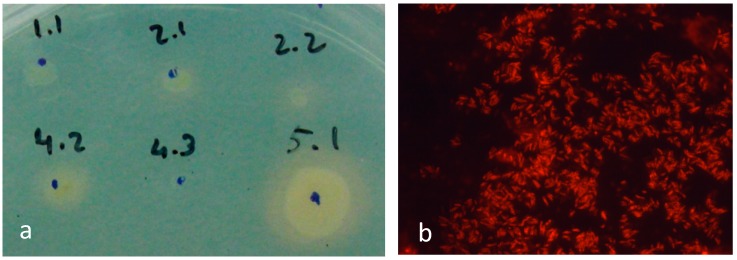
Production of siderophores and specialised lipids by bacteria. (**a**) CAS agar diffusion assay showing siderophore production (yellow halo) in the isolated *S. aureus* SA (marked as 1.1) and *Staphylococcus* sp. SH (indicated as 5.1); (**b**) *R. erythropolis* cells stained with Nile Red show storage lipids inside the cells and glycolipids excreted by the cells.

Both *S. aureus* SA and *Staphylococcus* sp. SH isolated from the cell phones produced siderophores when placed in iron depleted media (Figure 1a). Siderophores are low molecular weight compounds used by bacterial cells to sequester Fe^3+^ under conditions of iron depletion [18,19]. *Staphylococcus* strains have also several iron-uptake ABC transporters comprising membrane-anchored lipoproteins on the external surface of the membrane, transmembrane permease proteins, and ATPases that provide energy for the process [20,21].

**Figure 2:**
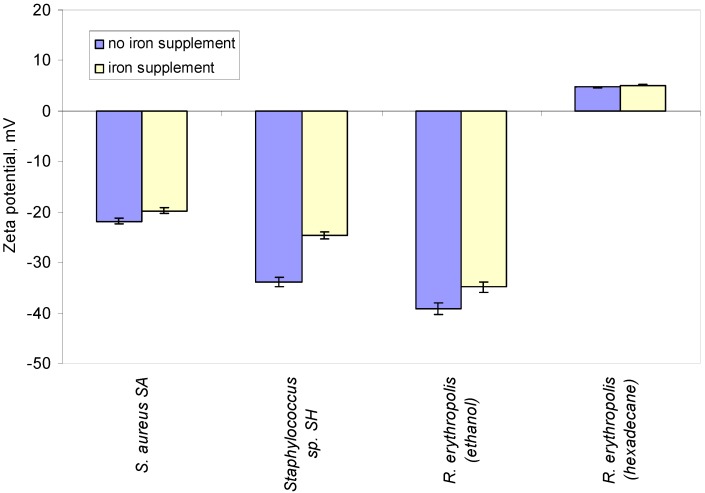
Influence of an iron supplement of 0.005 g/L iron(II) sulfate, in the growth media, on the zeta potential of the cells.

To test if the magnetic and electrostatic interactions between bacterial cells and metallic surfaces could be further enhanced, 0.005 g of iron per litre were added to the growth media of the bacteria. The cells grown in media supplemented with iron were compared to those grown on the same media without iron supplementation. Iron accumulation inside the cells was expected to stimulate their response to applied magnetic fields. In fact, non-crystalline magnetic inclusions have been reported in *Staphylococcus* species [22]. In the present study, the zeta potential of the cells, which may act as a measure of the surface charge of the cells, was influenced by both the carbon source used and concentration of iron(II) sulfate (Figure 2). When 0.005 g·L^−1^ of iron was supplemented to the growth media, the zeta potential of *S. aureus* SA decreased by 9.2% whilst that of *Staphylococcus* sp. SH decreased by 27.1%. A previous study showed a strong correlation between the chain length of n-alkanes used as carbon sources and the zeta potential of *R. erythropolis* DCL14 cells, with cells even becoming positive when grown on C14–C16 alkanes [23]. Changes in cell hydrophobicity and net surface charge that occurs during adaptation to the growth media affects the amount of cells that adhere to metallic and non-metallic surfaces [23,24,25].

In the current study, *R. erythropolis* DCL14 cells grown on ethanol exhibited a zeta potential 10% less negative when iron was added to the medium. However, these cells exhibited a zeta potential that was 8.5% more positive when grown with n-hexadecane as carbon source. Cells of strain DCL14 produced also glycolipids (Figure 1b), which acted as biosurfactants, thus decreasing the surface tension of the medium to 58.3 and 22.0 mN/m when the cells grew on ethanol and n-hexadecane, respectively. A decreased surface tension of the medium should aid the penetration of the cells in the defects.

### 2.3. Application of Magnetic and Electric Fields to Help Bacterial Distribution

Bacterial cells in suspension behave like solid particles. To achieve a homogeneous distribution of the cells on the surfaces of the samples to be analysed and better penetration into the defects, the movement of the cells in horizontal and vertical planes was enhanced by the application of magnetic and electric fields. Images taken with 10.4 s exposure enabled the determination of the distance travelled by the cells and the selection of those cells that travelled the longest distance.

**Figure 3:**
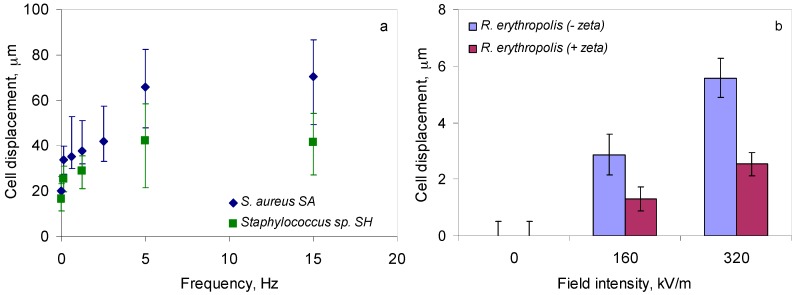
Cell displacement observed with an exposure of 10.4 s when *Staphylococcus* cells were placed on a glass surface subjected to magnetic fields (**a**) and when *R. erythropolis* cells presenting negative or positive zeta potential were placed under an electric field (**b**).

When exposed to magnetic fields created by electric currents with modified sine waves with frequencies of 1.25, 5 and 15 Hz, *S. aureus* SA cells travelled on average 38, 66, and 70 µm whilst *Staphylococcus* sp. SH cells travelled 29, 42, and 54 µm (Figure 3a). No significant differences (between 1.5% and 6.7%) were observed in the distance travelled by cells of either strain when modified sine waves with frequencies above 5 Hz were applied to create a magnetic field.

As previously mentioned, the zeta potential of *R. erythropolis* DCL14 cells can be influenced by the carbon source used for cell growth (Figure 2; [23]). When subjected to an electric field, cells grown on ethanol and presenting a negative zeta potential moved towards the anode, whilst cells grown on *n*-hexadecane and exhibiting a positive zeta potential moved towards the cathode. To favour the highest potential differences between cells and electrodes and the cell displacement, *R. erythropolis* cells were grown on ethanol without iron supplementation to obtain the highest negative zeta potential, whilst the medium used for cell growth using *n*-hexadecane as carbon source was supplemented with iron to increase the positive net surface charge of the cells (cf. Figure 2). The distance travelled by the cells was dependent on both their zeta potential and on the electric field applied: cells with negative net surface potentials were displaced 2.9 and 5.6 µm, whilst cells with positive potential were displaced 1.3 to 2.5 µm when electric field intensities of 160 and 320 kV/m were applied, respectively (Figure 3b). No displacement of the cells was observed in the absence of an electric field (Figure 3b).

### 2.4. Application of Bacterial Cells for the Identification of Standard Defects

A methodology for observing bacterial cells inside defects produced on the surface of metallic and polymeric materials was recently validated by our team [11,12]. It comprises the following steps:

- selection of the area to inspect;
- surface cleaning;
- deposition of bacterial suspension;
- penetration by and adherence of bacteria to defects;
- removal of the excess of bacterial suspension;
- inspection and evaluation of the surface using a microscope with fluorescent light;
- cleaning and sterilization of the material.

For validation purposes, after cleaning the surface and prior to the deposition of the bacterial suspension, the surface should be inspected under the microscope to ascertain that the surface is cell-free before the assays. Similarly, after cleaning and sterilization of the material at the end of the tests, the samples should be inspected under the microscope to assess if they are cell-free. Control samples should also be inspected by SEM.

**Figure 4:**
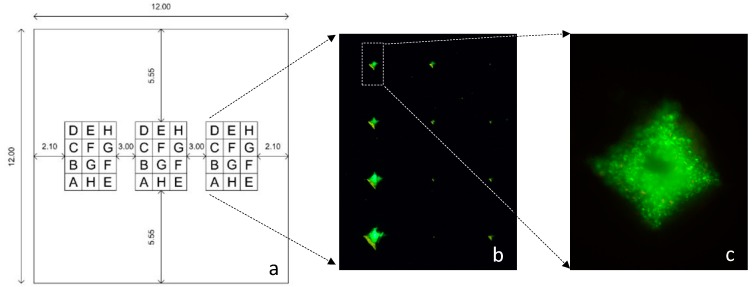
(**a**) Matrices of pyramidal defects created on the tested surfaces (units in mm). The letters represent the force applied to create the indentations: A—100 N, B—50 N, C—30 N, D—20 N; E—10 N; F—5 N; G—2.5 N and H—1 N. Units shown in mm; (**b**) Matrix of defects shown by the presence of green stained *R. erythropolis* cells; (**c**) Pyramidal defect filled with green stained cells.

To compare the ability of the bacterial cells to reveal each defect on the different metallic materials tested, 4 × 3 matrices containing surface indentations 300 µm apart were arranged in such a way that near a small defect there was always a larger one (Figure 4). The indentations were produced using a Vickers hardness testing machine from Mitutoyo with a square base pyramid with an angle between opposite faces of 136° (Figure 4a). Consequently, the depth of each pyramid is about 1/5 of its side length. This defect morphology presents a very low ratio of depth/open area, constituting one of the most adverse conditions in conventional dye penetrant NDT inspection, due to the possibility of removing the penetrant from the defect during the removal stage. The fluorescent dyes used allowed the easy observation of individual cells as they appeared quite bright over a dark field (Figure 4b,c).

The application of magnetic or electric fields in the horizontal direction may help the distribution of the cells across the surface to be analysed, whilst vertical fields may contribute to the entrance of the cells in the defects [12]. However, the fields may also influence the properties of the cells. It is known that cell–surface interactions involve e.g., hydrophobic and electrostatic interactions [26].

**Figure 5:**
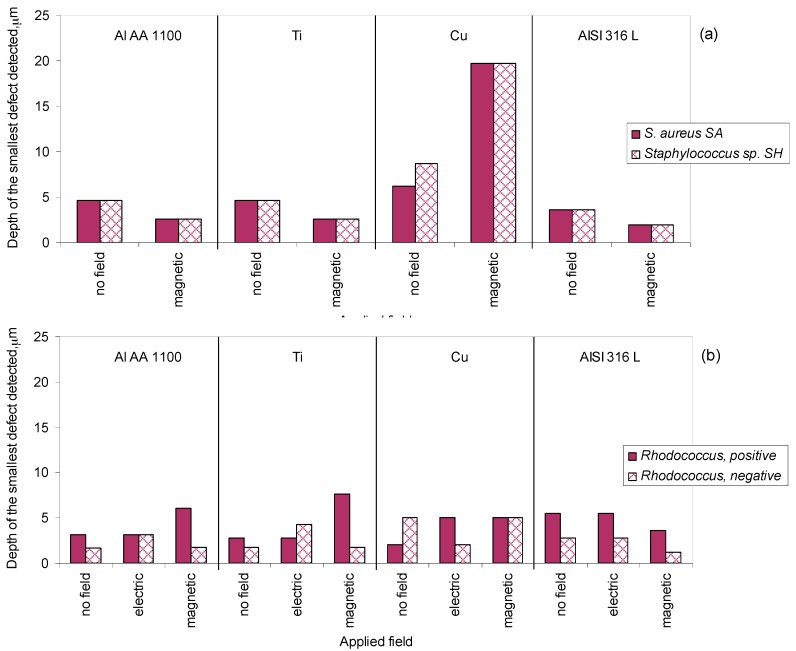
Influence of the applied field on the ability of the *Staphylococcus* (**a**) and *Rhodococcus* (**b**) cells to enter pyramidal indentations made as standard defects on metallic surfaces.

In the absence of an applied magnetic field, both *S. aureus* SA and *Staphylococcus* sp. SH helped the identification of the smaller defects in AISI 304L stainless steel (indentation pyramid with a depth of 3.7 µm) than in titanium or AA 1100 aluminium alloy (indentation pyramid with a depth of 4.6 µm; Figure 5). The application of a magnetic field in the 3 orthogonal spatial directions with 5 Hz allowed the entrance of both species in defects that were nearly half the size of those visible when no field was applied: in pyramidal defects with depths of 2.0 and 2.6 µm for steel and both titanium and aluminium, respectively. However, when the *Staphylococcus* cells were used to analyse defects made on copper samples, they only entered relatively large defects: with a side length of 30.3 µm and 6.2 µm depth when *S. aureus* SA was used, and a side length of 42.6 µm and 8.7 µm depth when cells of *Staphylococcus* sp. SH were applied (Figure 5). The application of a triaxial magnetic field with 5 Hz apparently further enhanced the known antimicrobial effect of copper [27,28]. With this metal, the *Staphylococcus* cells were only visible in the pyramids with a side length of 96.7 µm and depth of 19.7 µm.

In the absence of an applied electric field, *R. erythropolis* cells grown on *n*-hexadecane as carbon source, and presenting positive zeta potential, allowed the identification of smaller defects in copper (side length of 9.7 µm and depth of 2 µm) than cells grown on ethanol and with negative net surface charge (side length of 24.6 µm and depth of 5 µm; Figure 5). In all other surfaces tested, when no field was applied, *R. erythropolis* cells presenting a negative zeta potential entered defects smaller than their positive counterparts. The application of a horizontal and vertical electric field with 8 kV during the assays with *R. erythropolis* cells with positive zeta potential allowed the identification of the same defects as when no field was applied, except in copper samples where only pyramidal defects with sides longer than 14.6 µm were shown (Figure 5). The reverse was observed with cells with negative net surface charge: smaller defects could be observed in copper samples but the detectability of the defects was worse in aluminium and titanium samples as compared to when no field was applied (Figure 5).

To assess if the cells presenting different zeta potentials could also be influenced by magnetic fields, *R. erythropolis* cells were tested under a triaxial magnetic field with 5 Hz. This allowed the observation of the smallest defects made in AISI 304L stainless steel, with only 1.7 µm of depth, with cells of both positive and negative zeta potential (Figure 5). However, in the other metallic surfaces, no changes were observed with cells with negative net charge whilst positive cells entered only defects that were larger than those observed without applied fields. The results thus indicate that the properties of the cells could be influenced, affecting the interaction between cells and surface. The application of magnetic and electric fields assured that all indentation matrices made on the surfaces were covered by cell suspension, but the existence of cells inside the pyramidal indentations during the observation stage is dependent on (i) the entrance of cells into the defect and (ii) the permanence of the cells in the defect after the removal of the excess of the culture suspension. This depends on the strength of the forces between cells and surfaces.

### 2.5. Application of Bacterial Cells for the Identification of Real Defects

**Figure 6:**
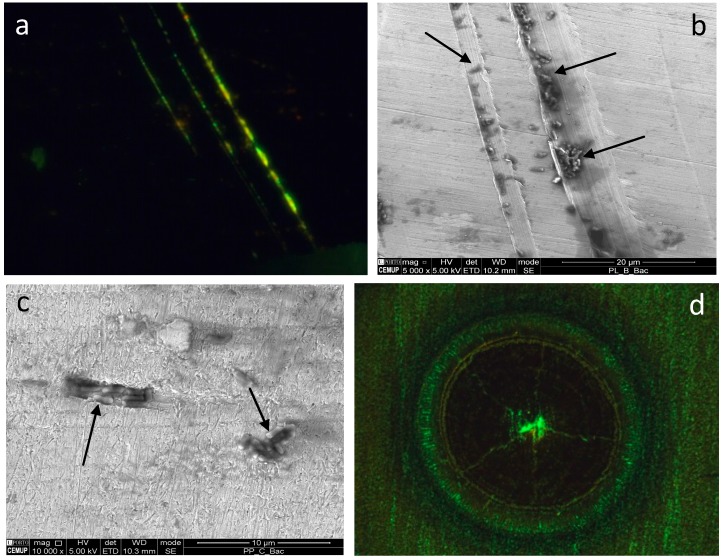
*Rhodococcus erythropolis* cells indicate the presence of scratches (**a**,**b**) created during the manipulation of microscrews for dental implants and voids (**c**) created during the manufacturing process by Micro Powder Injection Molding (µPIM). The cells were observed by fluorescence microscopy (**a**,**d**; magnification 30×) and SEM (**b**,**c**; magnification 5000× in **b** and 10,000× in **c**; some cells indicated by arrows). *R. erythropolis* stained with the green fluorescent dye SYTO^®^9 show radial cracks on a pulsed laser welding of titanium thin sheets (**d**).

The methodology proposed was not only efficient for identifying pyramidal indentations but also indicated scratches and other defects inadvertently introduced on the metal surfaces under study (Figure 6a–c). The cells could clearly indicate both the voids that were created on AISI 316L stainless steel microscrews made for dental implants during fabrication by Micro Powder Injection Molding (Figure 6c) and the scratches that were inadvertently made during manipulation of the microscrews in the laboratory (Figure 6a,b). Besides, the cells could efficiently show cracks formed on a pulsed laser weld of titanium thin sheets when stained with a fluorescent dye and observed under an optical microscope with fluorescent light (Figure 6d).

**Figure 7:**
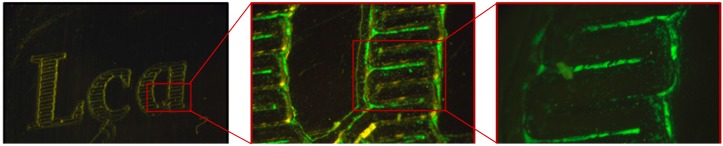
*Rhodococcus erythropolis* cells were able to highlight both the topography and roughness of a gold pendant (magnification from left to right: 30×; 150×; 300×).

When tested on engraved gold jewellery, the cells were able to accurately reveal both the topography and roughness of the sample, with the polished part of the surface remaining nearly cell free (Figure 7). Since the cells were deposited for less than 5 min on the surface of the metal samples to be examined, cell adhesion to the metal surfaces was reversible. Cleaning and disinfection of the samples could be achieved by any of the following methods: chemical sterilization, by application of 70% ethanol; irradiation with ultraviolet light (lamp Osram HNS 8 W G5, dominant wavelength 254 nm); and dry heat, or by placing the metallic samples in boiling water (data not shown). After the sterilization procedure, the samples were rinsed with distilled water, dried with tissue wipes, and observed under a microscope. No debris or whole cells were observed on the surfaces (data not shown).

**Figure 8:**
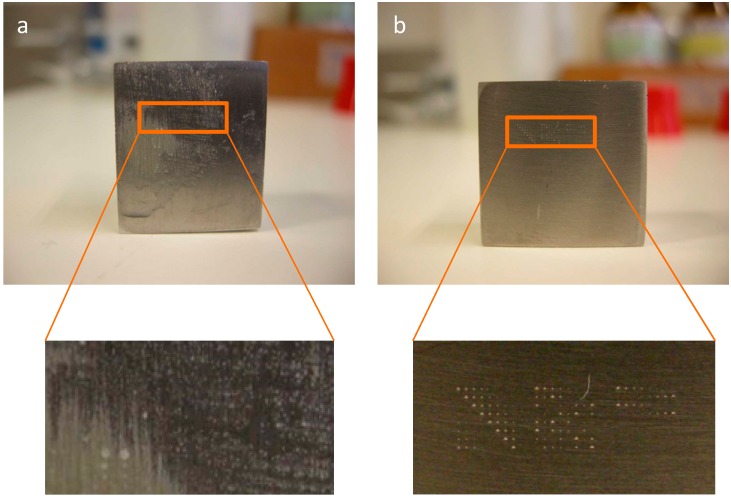
Colonies of *R. erythropolis* cells observed after letting the cells inside the defects grow overnight show scratches made by sanding (**a**) and matrices of indentations (**b**) under the naked eye.

To improve the stage of inspection and evaluation of the surface, and in particular to test the possibility of evaluating the surface under the naked eye, bacterial growth was promoted on the surfaces. It is known that bacterial cells may form biofilms on the surface of nearly all surfaces, including metals [25,29]. After the deposition stage, the excess liquid was removed and the sample was placed on sterile growth media overnight with temperature and agitation control. The cells inside the defects multiplied, allowing the observation of colonies under the naked eye which showed scratches made by sanding (Figure 8a) and matrices of defects made (Figure 8b).

## 3. Discussion

Non-destructive Testing (NDT) techniques for the identification of defects occurring during microfabrication of microstructures and microsystems are of paramount importance, especially following their application in e.g., biological, medical, and aeronautical systems. In the present study, we propose to push the frontier of NDT by introducing the use of biological phenomena in the scope of NDT techniques, showing the application of bacterial cells to help the visualisation of micro surface defects. In a previous validation study [11], the *R. erythropolis* cells were simply deposited on the surface to be analysed and the coverage of the whole surface was dependent on the concentration of cells in the suspension. Since most bacterial species that were good candidates for this new NDT methodology do not possess locomotion structures such as flagella or pili, we tested the hypothesis of promoting cell movement over the surfaces to be tested by the application of magnetic or electric fields. At the beginning of this study it was already known that the net surface charge of *R. erythropolis* DCL14 cells could be influenced by the carbon source used for growth [23], making this strain a good candidate to be tested in the assays involving electric fields. To find bacterial strains responsive to magnetic fields, we isolated bacteria from samples collected from mobile phones and found two *Staphylococcus* strains able to move over significant distances in a magnetic field when compared to their size.

The phenotypic characteristics of both *S. aureus* SA and *Staphylococcus* sp*.* SH, including the small size (around 600 nm) and the ability to move in magnetic fields aided the penetration of the cells into small defects. The unexpected response of these cells to the imposed magnetic fields could result from the capture of iron by siderophores and especially by lipoproteins positioned at the external surface of the cellular membrane. Besides, non-crystalline magnetic inclusions previously found in *Staphylococcus* sp. SH have been shown to enable movement of the cells, which could be brought about by magnetic fields [22].

Staphylococcal lipoproteins play a role in the acquisition of iron by serving as binding proteins for both heme and transferring iron, and as siderophore-binding components of ABC transporters [21]. Thus, when the Mueller–Hinton medium (iron concentration ca. 0.5–0.8 mg·L^−1^ [30]) was supplemented with additional 0.005 g of FeSO_4_·7H_2_O per litre (iron added 1.0 mg·L^−1^), the zeta potential of the cells decreased, suggesting that iron ions could be at the surface resulting in a less negative character of the cell surface. Furthermore, ferritin proteins have been identified in *Staphylococcus* species and shown to function as iron storage proteins [31]. When studying freshwater magnetotatic bacteria, Frankel and co-workers observed that besides magnetite, there was a substance that produced an extra quadrupole doublet which the authors hypothesised could result, among others, from ferritin, which at that time had not been recognized in prokaryotic cells [32]. In order to study iron-binding centres in bacterial ferritins, Pereira et al. showed that in the wild type of *Desulfovibrio vugaris* a diamagnetic diferrous species, a mixed valence Fe^2+^Fe^3+^ species and a mononuclear Fe^2+^ species were present, but a paramagnetic diferrous species was identified in a variant of the bacterium [33]. Although ferritin can only bind relatively small amounts of iron (a cell might bind ca. 7000 molecules of ferritin, containing a total of ca. 10^7^ atoms of iron) and therefore the cells should be weakly paramagnetic, ferritin labelled particles (including polyacrylamide beads with size similar to whole cells) could be magnetically removed from a flowing suspension [34].

In the present study, the average distance travelled by *Staphylococcus* sp. SH and especially *S. aureus* SA cells when magnetic fields were applied was considerable when compared to their cell diameter (Figure 3a). *R. erythropolis* DCL14 cells, exhibiting both negative and positive zeta potentials, moved only 0.6–2.5 times the length of the cell when under an electric field (Figure 3b). Nevertheless, the horizontal and vibrational movement of the cells over the surface of the samples containing the indentations allows a homogeneous distribution of the cells over the area to be inspected. Other cell properties, including the ability of *R. erythropolis* to produce biosurfactants which lowers the surface tension of the medium, also helps the penetration of the cells by capillarity into the defects. Capillarity, together with wetting-contact angle, is of paramount importance in tests using liquid penetrants and is usually achieved by environmentally toxic chemical compounds [35]. Handling and disposal costs of synthetic and petroleum based oils used as carrier fluids for the fluorescent dye penetrants used in non-destructive inspections have been estimated at a few million dollars per year [36]. The proposed method using bacterial cells could thus constitute a cleaner and cheaper alternative to the existing liquid penetrants.

According to the extended Derjaguin–Landau–Verwey–Overbeek (DLVO) theory of colloid stability, microbial adhesion is governed by Lifshitz–Van der Waals forces, electrostatic attraction/repulsion, Lewis acid–base interactions and Brownian motion forces [15,26]. Since in the present study irreversible adhesion of cells to surfaces was prevented to maintain the non-destructive character of the procedure, the cells could only be observed inside the pyramidal defects if they could resist the hydraulic shear force caused by the cleaning of the surfaces prior to the observation under the microscope. The adhesion forces of bacteria to metals are influenced by electrostatic interactions and metal surface hydrophobicity, which in artificial sea water present the following zeta potential (in mV): Al—1754.0 ± 29.4; stainless steel—1364.4 ± 28.6; copper—449.4 ± 19.3 [37]. *Staphylococcus* sp. SH presented a more negative character (zeta potential of −33.9 mV) than *S. aureus* (zeta potential of −21.8 mV), but the same defects could be identified using both strains (Figure 5). The poor results observed with copper could result from lower cell adhesion forces due to its lower zeta potential which could lead to cells being wiped from the defects during the cleaning procedure. Although the exposure time during the assays was relatively short, the antimicrobial effect of metallic copper is also well known [27], and could have also influenced cell behaviour. The properties of the metals apparently had a higher influence on cell adhesion when magnetic and electric fields were applied to *R. erythropolis* cells and no general rule may be drawn (Figure 5). However, in general, smaller defects could be observed with *Rhodococcus* than with *Staphylococcus* cells.

The examples presented further demonstrate the feasibility of using bacterial cells on real samples made from different metals by different techniques as a valuable NDT to identify micro defects. From microscrews for dental implants to pulsed laser welding and engraved jewels, bacterial cells may be used to help the identification of defects produced during microfabrication. Furthermore, the cells also revealed very small scratches and holes that were inadvertently introduced on the metal surfaces of the samples during their manipulation. This suggests that further study could improve the detection limit of the technique in micro-manufactured and non-planar components (3D complex geometries) in different materials. The detection of micro and nano surface defects is a challenging area of NDT and is of paramount importance in current and arising applications such as transparent ceramics for optical applications and solar wafers where surface may also present an aesthetic, optical, or tribologic role.

One last comment goes to biosafety issues related to the procedure. *R. erythropolis* cells belong to biosafety level 1, and so standard microbiological practices are sufficient and the tests can be performed on an open laboratory bench. Although *Staphylococcus* species are part of the human flora (e.g., *S. aureus* is primarily found in the nose and on the skin of human beings), they belong to biosafety level 2, which includes microbes that are indigenous but associated with diseases of varying severity. If proper microbiological practices are followed, these microbes can be safely used in an open laboratory bench, as long as the potential for producing splashes or aerosols is low. Under such conditions, the use of laboratory coats and gloves as protective measures should be sufficient. At the beginning of this work, we tested the tolerance of both strains and found them tolerant to most commonly used antibiotics (data not shown). Currently, we are testing these bacterial strains in a lyophilised and dried state, and we are also testing commercially available level 1 bacteria known to respond to magnetic fields. It is envisaged that the cells may be provided in kits containing an appropriate medium, dye(s), and a disinfectant to be handled by technicians without microbiological training.

## 4. Materials and Methods

### 4.1. Strains and Bacterial Growth

Two *Staphylococcus* strains were isolated from samples collected from the space between the battery and the circuit board of mobile phones using sterile cotton swabs. The cells were screened for their ability to move in response to a magnetic field and were identified using the Sherlock^®^ Microbial ID System (MIDI, Inc., Newark, DE, USA), as previously described [38,39]. In summary, tryptic soy agar plates containing each isolate were grown at 30 °C for 24 ± 1 h, after which the exponentially growing cells were harvested, and their fatty acids were extracted and methylated to fatty acid methyl esters (FAMEs) using the Instant FAME procedure according to the instructions provided by MIDI. FAMEs were analysed on a 6890N gas chromatograph from Agilent Technologies (Palo Alto, CA, USA).

The two isolates were also identified by 16S ribosomal RNA gene sequence analysis. The 16S rRNA-encoding genes of the two bacterial strains were determined according to previously described basic protocols [40]. Briefly, the genomic DNA was extracted from a 1 mL aliquot of an overnight grown culture using the commercial kit “High Pure PCR Template Preparation Kit” (Roche, Meylan, France), according to the manufacturer’s instructions. The DNA concentration was estimated by assessing the absorbance at 260 nm, in a NanoDrop ND 1000 spectrophotometer (Thermo Fisher Scientific, Waltham, MA, USA). To amplify the 16S rRNA-encoding genes, a polymerase chain reaction (PCR) was performed with the Eubacteria universal primer pair E334F (5′CCAGACTCCTACGGGAGGCAGC3′) and E939R (5′ CTTGTGCGGGCCCCCGTCAATTC3′) [41]. Amplification mixtures were prepared in a total volume of 50 μL, containing 50 ng of total DNA as template, 200 μM of each deoxynucleotide, 0.5 μM of each primer, 1.5 mM MgCl_2_, and 1U of Taq polymerase (Citomed). PCR conditions included an initial step of DNA denaturation at 95 °C for 5 min, followed by 30 cycles of 95 °C for 45 s, 58.2 °C for 60 s, and 72 °C for 45 s, and a final extension step at 72 °C for 7 min. Amplified fragments were visualised after electrophoresis in 0.8% (*w/v*) agarose gels and purified using the NZYGelpure kit (NZYTech, Lisbon, Portugal), according to the manufacturer’s instructions. The DNA fragments were sequenced as a paid service by Eurofins MWG Operon (Ebersberg, Germany). The obtained sequences were aligned and compared with homologous sequences deposited in GenBank using the Basic Alignment Search Tool (BLAST, Bethesda MD, USA) [42].

For the different assays, the *Staphylococcus* cells were grown in Mueller–Hinton broth at 37 °C and 200 rpm in an Agitorb 200 (Aralab, Rio de Mouro, Portugal) incubator and collected by the end of the exponential phase.

*Rhodococcus erythropolis* DCL14 was isolated by the Division of Industrial Microbiology of the Wageningen University, in The Netherlands [43]. It is stored and maintained at iBB, IST, Portugal. The cells in the present study were grown in mineral medium [44] with 0.25% absolute ethanol or *n*-hexadecane as sole carbon sources at 28 °C and 200 rpm, and collected by the end of the exponential phase. When necessary (stated in the text), the mineral medium was supplemented with additional 0.005 g·L^−1^ of FeSO_4_·7H_2_O, to reach a final concentration of 0.01 g·L^−1^.

### 4.2. Zeta Potential

The bacterial cells collected during the exponential phase were washed 3 times and suspended in 10 mM KNO_3_. The zeta potential was calculated from the electrophoretic mobility (according to the method of Helmholtz-von Smoluchowski [45]) using a Doppler electrophoretic light scattering analyser (Zetasizer Nano ZS, Malvern Instruments Ltd., Worcestershire, UK). Calculations were made automatically using the Zetasizer Software 7.03, from Malvern Instruments Ltd.

### 4.3. Detection of Siderophores

Siderophore production was assessed in CAS agar plates, prepared according to Schwyn and Neilands [46], by inoculating the stock bacteria with the aid of a sterilised toothpick. The plates were incubated at 30 °C and monitored by image analysis every 24 h.

### 4.4. Surface Tension of Culture Supernatants

The surface tension of cell-free culture supernatants was measured by a plate device according to the Wilhelmy technique [47], using a K8 tensiometer from Krüss GmbH (Hamburg, Germany).

### 4.5. Application of Magnetic Fields under the Microscope

A customised functional prototype was developed to produce magnetic fields with peak intensities in horizontal and vertical directions, respectively. The magnetic fields were produced by a solenoid from a stator of a stepper motor when excited by an AC electrical current with modified sine wave at frequencies between 0.125 and 15 Hz. The stator was placed on the stage of an Olympus CX40 microscope (Olympus, Tokyo, Japan).

The cells were stained with a LIVE/DEAD^®^ BacLight™ Bacterial Viability Kit from Molecular Probes (Life Technologies, Thermo Fisher Scientific, Waltham, MA, USA) containing a mixture of SYTO^®^9 green fluorescent nucleic acid stain (which stains all bacteria) and propidium iodide (which stains red only bacteria with damaged membranes). Cells were observed under fluorescent light provided by an Olympus U-RFL-T burner and an U-MWB mirror cube unit (excitation filter: BP450-480; barrier filter: BA515; Olympus, Tokyo, Japan) placed on the microscope.

To compare the movement of the different bacterial cells under the magnetic fields applied, photographs were captured during 10.4 s of exposure by an Evolution™ MP5.1 CCD colour camera using software Image-Pro Plus, both from Media Cybernetics, Inc. (Rockville, MD, USA). The distance traveled by each cell was calculated using Image-Pro Plus version 6.0. The images were calibrated using the TetraSpeck™ Fluorescent Microspheres Sampler Kit from Molecular Probes^®^ (Life Technologies, Thermo Fisher Scientific, Waltham, MA, USA), which contains 0.1, 0.5, and 4.0 µm TetraSpeck™ beads.

### 4.6. Application of Electric Fields under the Microscope

A variable DC high voltage power supply was used to create electric fields between two parallel copper plates separated by 25 mm. Eight kV were applied between the plates, creating a constant electric field with an intensity of ca. 320 kV/m. The movement of the cells was followed as described for the magnetic fields.
